# Is gallbladder PoCUS diagnostic accuracy accessible to medical students after PoCUS training exclusively on healthy volunteers? A pilot randomized control trial

**DOI:** 10.1186/s13089-023-00317-6

**Published:** 2023-04-10

**Authors:** Florence Dupriez, Bastian Rodrigues de Castro, Félix Gendebien, Antoine Fasseaux, Matthieu Gensburger, Laurent Marissiaux, Andrea Penaloza, Xavier Bobbia, Robert Jarman

**Affiliations:** 1 Emergency Department, Cliniques Universitaires Saint Luc, Av Hippocrate 10, 1200 Brussels, Belgium; 2 Emergency Department, Hôpital de Jolimont - Lobbes, Lobbes, Belgium; 3 Emergency Department, Hôpital de Jolimont - Lobbes, Haine-Saint-Paul, Belgium; 4 Emergency Department, Centre Hospitalier Universitaire de Liège, Liège, Belgium; 5 Emergency Department, CHU Montpellier, Montpellier, France; 6 Emergency Department, Royal Victoria Infirmary, Newcastle, UK

**Keywords:** Point-of-care ultrasound, Clinical ultrasound, Curriculum, Ultrasound teaching

## Abstract

**Background:**

Point-of-care ultrasound (PoCUS) is increasingly used in clinical practice and is now included in many undergraduate curricula. Here, we aimed to determine whether medical students who participated in a PoCUS teaching program with several practical training sessions involving healthy volunteers could achieve a good level of diagnostic accuracy in identifying gallbladder pathologies. The intervention group (IG) was trained exclusively on volunteers with a healthy gallbladder, whereas the control group (CG) had access to volunteers with a pathological gallbladder as recommended in most PoCUS curricula.

**Materials and methods:**

Twenty medical students were randomly assigned to the IG and CG. After completing the training program over 2 months, students were evaluated by three independent examiners. Students and examiners were blind to group allocation and study outcome. Sensitivity and specificity of students’ PoCUS gallstone diagnosis were assessed. Secondary outcomes were students’ confidence, image quality, acquisition time, and PoCUS skills.

**Results:**

Sensitivity and specificity for gallstone diagnosis were, respectively, 0.85 and 0.97 in the IG and 0.80 and 0.83 in the CG. Areas under the curve (AUC) based on the receiver operating characteristic curve analysis were 0.91 and 0.82 in the IG and CG, respectively, with no significant difference (*p* = 0.271) and an AUC difference of -0.092. No significant between-group difference was found for the secondary outcomes.

**Conclusions:**

Our pilot study showed that medical students can develop PoCUS diagnostic accuracy after training on healthy volunteers. If these findings are confirmed in a larger sample, this could favor the delivery of large practical teaching sessions without the need to include patients with pathology, thus facilitating PoCUS training for students.

**Supplementary Information:**

The online version contains supplementary material available at 10.1186/s13089-023-00317-6.

## Background

Many medical specialties and paramedical fields are increasingly using point-of-care ultrasound (PoCUS) [1–3]. As a pillar of clinical evaluation along with inspection, palpation, percussion, and auscultation [4, 5], PoCUS has now become essential in daily clinical practice, as it enhances differential diagnosis [6–8].

Medical schools have progressively introduced PoCUS into their curricula. In a position statement published in 2015, the American Academy of Emergency Medicine followed by the European Federation of Societies for Ultrasound in Medicine and Biology in 2016 recommended including PoCUS in the curricula of the main medical schools to improve the learning of core concepts and to enhance students’ understanding of physical examinations [4, 5]. In 2019, a position statement from the Alliance of Academic Internal Medicine also supported including PoCUS as part of medical school teaching [9]. A recent questionnaire reported that 72.6% of medical schools in the United States had an active PoCUS curricula [6], whereas a similar European study found PoCUS curricula in 40 out of 53 (75%) of the contacted universities [7]. Although medical students’ needs in terms of PoCUS are probably similar around the world, both studies highlight the heterogeneity of PoCUS teaching for medical students [7, 8]. Despite the ambitious goal for all physicians to learn to use PoCUS, there is limited literature about how to teach PoCUS both widely and efficiently.

At present, the postgraduate PoCUS curricula combine a theoretical introduction with practical exercises based on ultrasound image acquisition and interpretation on volunteers in order to collect normal and pathological PoCUS images in a logbook [10]. Experience is often gained in a clinical setting. Training in a clinical environment is nevertheless associated with several inconveniences. Indeed, the stress of the clinical setting can create real obstacles in gaining PoCUS skills, as the environment is driven by patients’ needs rather than medical students’ education. Bedside PoCUS supervision can further be complicated by the unavailability of supervisors and high patient flow [11]. The rise in the demand for POCUS training will inevitably increase the need for supervision, thus progressively limiting the current training method. It is therefore time to rethink the teaching of PoCUS before students begin clinical rotations, which would consequently involve teaching all undergraduate medical students.

In a clinical setting, PoCUS can be used in many ways during physical examinations. For abdominal assessments, most scientific societies encourage its use to address a specific clinical question rather than to provide a diagnosis, which is usually confirmed by a comprehensive ultrasound in radiology [5, 12, 13]. In daily practice, abdominal pain accounts for 7% to 10% of emergency department consultations [8], while the mean reported prevalence of abdominal pain in family physician consultations is 2.8% according to a recent systematic review [14]. In 7.7% of patients suffering from abdominal pain, the cause is biliary colic or cholecystitis [8]. The presence of gallstones in PoCUS is an important indication that points the physician to these two diagnoses. Although described in many curricula as an advanced PoCUS skill [10, 15, 16], gallbladder evaluations with PoCUS can truly enhance bedside differential diagnoses [12, 13, 17].

The main objective of this pilot randomized control trial was to evaluate the diagnostic accuracy of medical students for gallbladder assessment using PoCUS in terms of sensitivity and specificity. Students were divided into two groups: an intervention group (IG) that was trained exclusively on healthy volunteers with a normal gallbladder and a control group (CG) that additionally had access to volunteers with pathological gallstones as recommended in most PoCUS curricula. Students’ confidence about their diagnosis and ultrasound use were also evaluated as secondary outcomes. The other secondary objectives were image quality, students’ machine use, and acquisition time.

## Method

### Study design and ethics

This prospective pilot study was based on a double-blind randomized controlled model approved by the institutional review board (Comité d’éthique Hospitalo-Facultaire Saint Luc-UCL) of a tertiary university hospital in Belgium. The study did not involve any patients. The approval of the ethics committee was therefore consultative, and no registration number was given.

This paper was written according to the CONSORT guidelines. The study protocol was previously registered under clinical trials.gov (NCT04879459).

### Study timeline

**Fig. 1:**
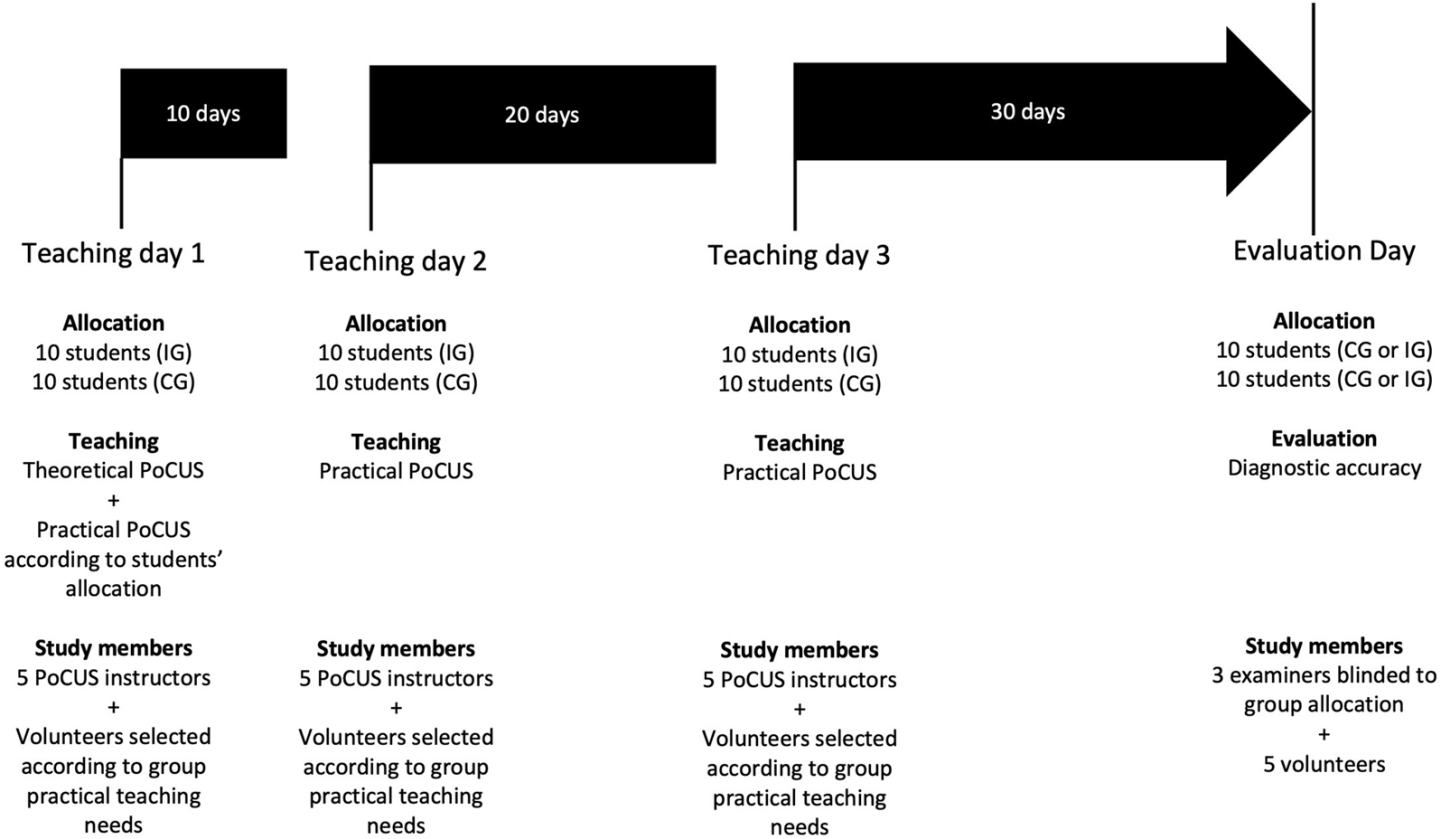
Study timeline. IG: intervention Group. CG: control group

The study timeline (Fig. 1) depicts the interaction between the study population and the study members.

### Study population

Fifth-year medical students enrolled at the Université Catholique de Louvain in Brussels, Belgium, were invited by email to participate in this PoCUS study. The first 20 eligible students who responded were selected. Students were eligible if they were PoCUS naïve and available on the four chosen study days between October 12 and December 10, 2021 (Fig. 1). Prior to inclusion, students signed an informed consent and a non-disclosure agreement written in French. According to the non-disclosure agreement, participants could not disclose the study to any third party for the entire study period; any breach would lead to their exclusion. Subsequently, the 20 students were randomly assigned to the two predefined groups of 10 students using a computed randomization (Randomizer for Clinical Trial Lite, Medsharing©, Fontenay-Sous-Bois, France).

The 10 CG students received gallbladder PoCUS training in accordance with the current recommendations [10, 18, 19] as well as a publication about learning curves [20]. They learned PoCUS on a mixture of volunteers with healthy and pathological (gallstones/polyps) gallbladders. The 10 IG students underwent gallbladder training exclusively on volunteers presenting a normal gallbladder. Students were blind to group allocation and study outcomes for the entire study period.

### Study members

#### Teaching faculty

Five instructors participated in the three teaching days. All instructors were emergency physicians certified in PoCUS use for gallbladders. Two instructors had validated an interuniversity diploma in PoCUS at University Paris V–Descartes (France), one had a Master’s degree in medical ultrasound from Teesside University (UK), one had a certificate in medical ultrasound from Teesside University (UK), and one had an advanced POCUS certificate from Université Libre de Bruxelles (Belgium). Both groups were taught by the same instructors who were not blind to student allocation. All instructors signed a non-disclosure agreement.

#### Volunteers

A total of 43 volunteers standardized patients (scanning models) were invited to participate in the study. Healthy volunteers were recruited by email, while volunteers with gallstones were recruited during surgical consultation for elective cholecystectomy or when diagnosed with gallstones during gallbladder ultrasound sessions organized for healthy volunteers before the study. Volunteers were eligible if they were aged over 17 years, had no persistent abdominal pain, and signed the informed consent form and non-disclosure agreement. All volunteers underwent a comprehensive gallbladder ultrasound in radiology at most 1 month prior to the start of the study to determine their gallbladder status (normal or gallstones and/or sludge). Volunteers, 17 male (40%) and 26 female (60%), were paired for body mass index (BMI) (+/−1) and age (+/− 5 years) between groups. Their characteristics are described in Table 1. All volunteers fasted for at least 3 h prior to the gallbladder scan. They were not aware of the study objectives or methodology. A total of 38 (88%) volunteers were allocated to the IG or CG for the teaching sessions depending on their gallbladder status and general characteristics. Among the 38 volunteers, 7 (18%) had a pathological gallbladder (gallstones or polyps) and participated exclusively in the CG teaching days. Five other volunteers participated in the evaluation day.

**Table 1:** Characteristics of volunteers

Volunteers	Intervention group	Control group	*p-*value
Mean age years (SD)	50 (+/−15)	47 (+/−15)	0.413
Weight kilograms (SD)	72.33 (+/−14)	79.53 (+/−23)	0.151
Height meters (SD)	1.69 (+/−0.08)	1.70 (+/−0.1)	0.824
BMI (SD)	25.22 (+/−4.5)	27.29 (+/−6.3)	0.147

BMI: body mass index; SD: standard deviation

#### Assessment faculty

Three examiners from different specialties (surgeon, radiologist, and emergency physician) certified in gallbladder ultrasound use and blind to students’ allocation participated in the evaluation. They were independent of the study and did not work in the university conducting the research; they did not contribute to the study protocol or participate in the theoretical and practical PoCUS teaching sessions. Two Belgian examiners spoke French, while the third examiner was from the UK and spoke English.

### Study setting

#### Teaching days

Teaching was organized on separate but consecutive days for the IG and CG to prevent interaction between groups and thus respect the non-disclosure agreement. On teaching day 1, students from both groups attended a 1-h theoretical session that included a web-based video in French (https://youtu.be/7ZqP2mKNOQg) about gallbladder PoCUS use with images and loops of normal and pathological (gallstones or sludge/polyps) gallbladders. The theoretical session focused on ultrasound machine settings (curvilinear probe, abdominal preset, depth, and gain), gallbladder recognition using POCUS, description of normal gallbladders, identification of gallstones, difference between gallstones and polyps, and description of POCUS pitfalls (duodenum, collapsed gallbladder). Students also participated in three 3-h practical teaching sessions (teaching days 1, 2, and 3) using a bedside Vscan Air™ (GE VINGMED Ultrasound AS, Horten, Norway) portable ultrasound probe connected to an iPad. Gallbladders were assessed using an abdominal setting with the curvilinear probe. The practical teaching days were distributed according to an expanding retrieval practice for effective learning, as supported by a recent review showing that expanding intervals between teaching sessions, as opposed to regular intervals, enhance long-term retention [21]. All students performed a total of 45 gallbladder PoCUS, with 15 being completed in each 3-h teaching session repeated across 3 teaching days. In the IG, all 45 (100%) of the gallbladder PoCUS performed by students were normal. In the CG, 24 (53%) of the gallbladder PoCUS were normal and 21 (47%) were pathological. Among the 21 pathological PoCUS, 18 (86%) showed gallstones and 3 (14%) polyps. The rotation of students between instructors was organized to ensure that all students underwent the same PoCUS training. During the 3-h session, students had access to one-on-one teaching at the bedside of volunteers. Every 10 min, the students changed places to perform the gallbladder PoCUS on another volunteer supervised by another instructor. During each 3-h session, the students performed a gallbladder PoCUS on five different volunteers and rotated three times, thus corresponding to a total of 15 PoCUS performed by each student. For the practical training, instructors used a teaching chart with relevant PoCUS information that was given to students at each practical session (Additional file 1—translated from French). At the end of the third teaching day, students from both groups were randomly assigned for the 30-min evaluation.

#### Evaluation day

**Fig. 2:**
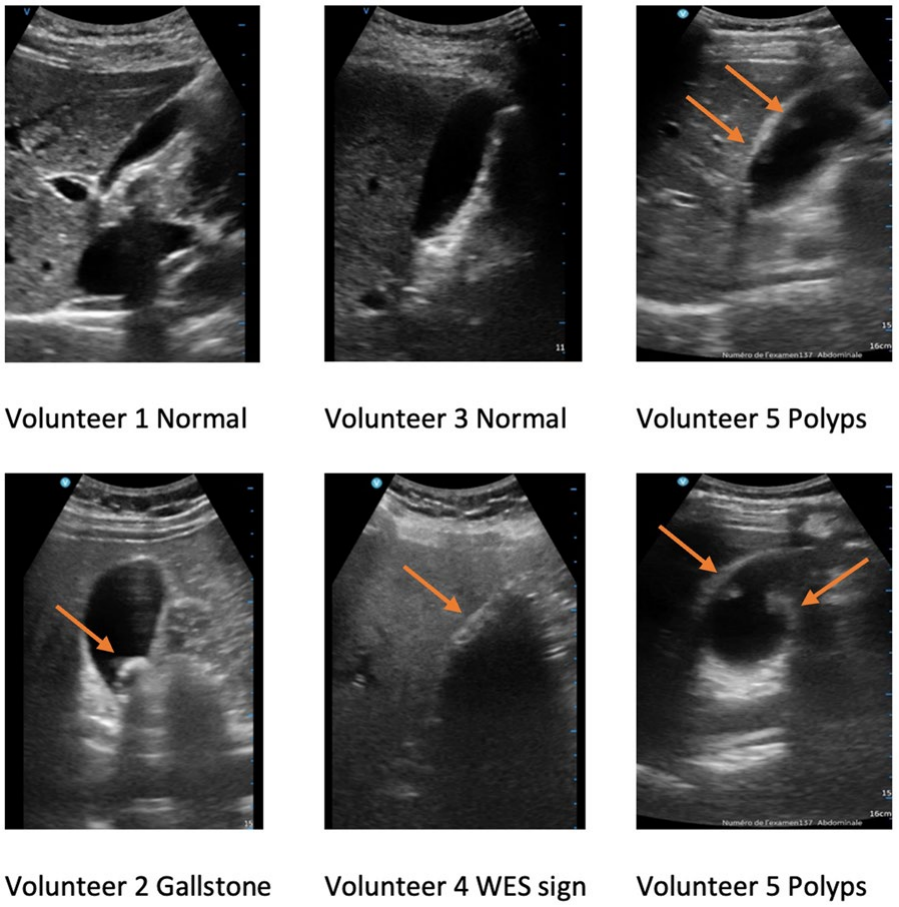
Gallbladder ultrasound images from the evaluation day. WES: wall-echo-shadow

Sensitivity and specificity for gallstone diagnosis by students using PoCUS was assessed on five volunteers: two had gallstones, two had a normal gallbladder, and one had polyps. Every 30 min, in front of the three examiners, a student was scheduled to perform five gallbladder PoCUS on the five volunteers set up in different rooms. Gallbladder ultrasound images are shown in Fig. 2 following the sequence of evaluations. During the entire evaluation, students were asked not to interact with the examiners. They could nonetheless speak with the volunteers and ask them to change position or take a deep breath. Students completed a form with their diagnosis (gallstones and/or sludge) and then provided a final diagnosis (normal/gallstones/polyps) after performing the PoCUS. Students’ confidence about their diagnosis and machine use was evaluated using a five-point Likert scale. The examiners who were aware of the volunteers’ gallbladder status were asked to fill in a form assessing image quality and quality of machine use. Examiners classified the PoCUS images obtained by students as interpretable or uninterpretable and assessed their machine use as satisfactory or unsatisfactory in terms of their ability to handle the probe with one hand and to properly manage gain and depth with the other. The students were unable to share their results until the end of the evaluation. The examiners were blind to the students’ diagnosis and confidence about their diagnosis and machine use.

### Outcomes

The main outcome of this study was the sensitivity and specificity of gallstone diagnosis by students using PoCUS. Sensitivity and specificity were explored in each group. As secondary outcomes, students’ confidence in their diagnosis and machine use was also reported, as were the examiners’ evaluation of the image quality and PoCUS use. The acquisition time was also evaluated in the IG and CG.

### Data management

Corresponding groups and names were kept in a secure database. Except for the study coordinator, no other investigator had access to the data. A number was randomly assigned to each participant and a letter to each group allocation to ensure blind analysis.

### Statistical analysis

Statistical analysis was performed with SPSS 26.0® and JMP pro16.0.0®. The primary outcome was evaluated using sensitivity and specificity estimates with a 95% confidence interval (CI) and the area under the curve (AUC) calculated based on receiver operating characteristic (ROC) curve analysis for the IG and CG. Regarding the secondary outcomes, a Mann–Whitney–Wilcoxon test was used to assess between-group differences in students’ confidence about their diagnosis and machine use according to a Likert scale. A Mann–Whitney–Wilcoxon test was also used to assess the between-group difference in the time taken for image acquisition. A nonparametric test was chosen due to the relatively small sample sizes and the potential skewness of the distribution of acquisition times. A proportion test was used to represent the examiner-assessed image quality and machine use of both groups. A *p*-value of < 5% corrected for multiple tests was considered significant.

As this is a pilot study, sample size calculation could not be performed due to the lack of previous evidence. We included 10 participants in each group due to the expected low attrition rate and the study feasibility [22].

## Results

**Fig. 3:**
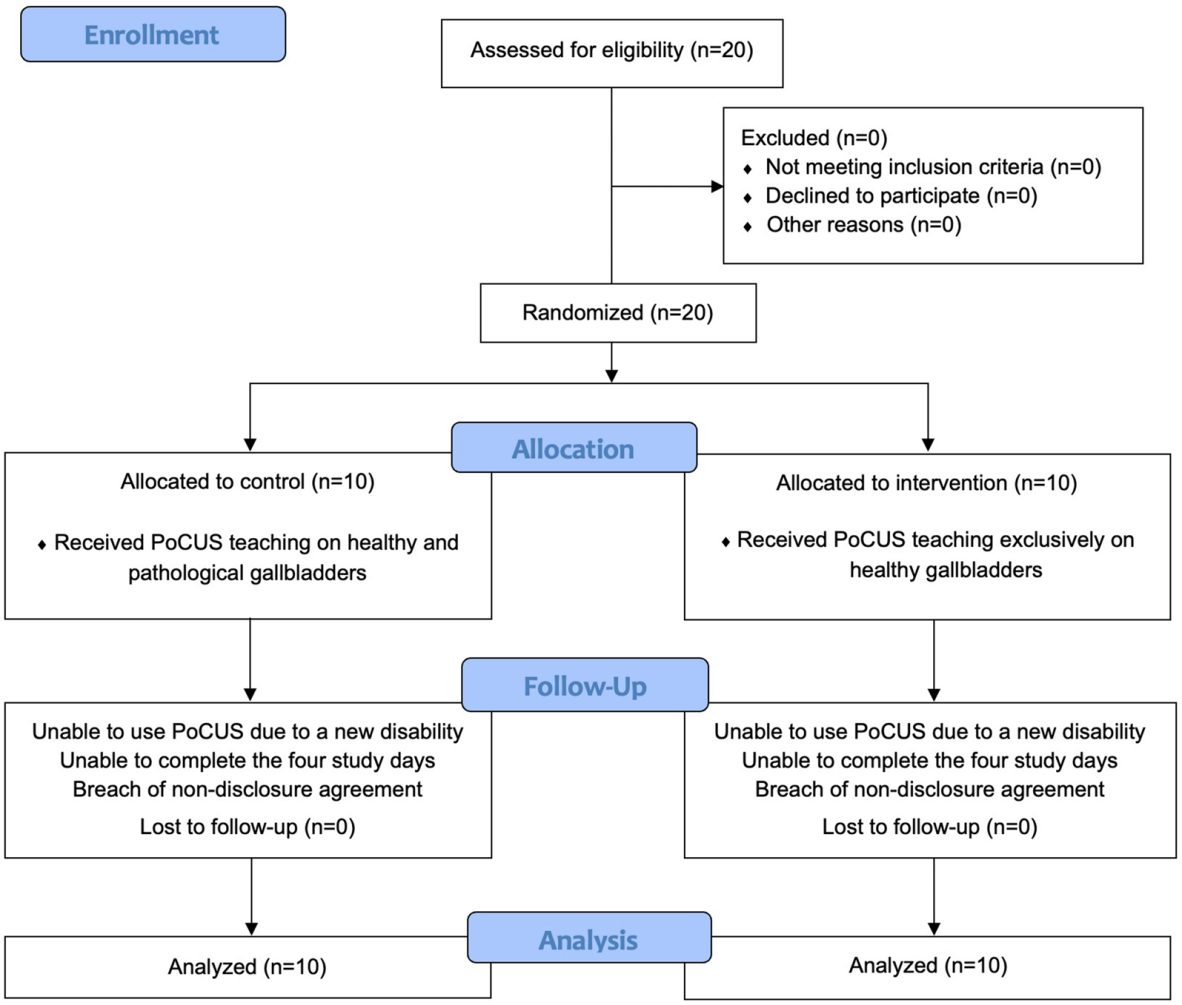
Consort flow diagram

Figure 3 depicts the CONSORT flow diagram. Sensitivity and specificity for the diagnosis of gallstones were, respectively, 0.85 (95% CI 0.62–0.97) and 0.97 (95% CI 0.83–1) in the IG and 0.80 (95% CI 0.56–0.94) and 0.83 (95% CI 0.65–0.94) in the CG. Calculated AUC were 0.91 and 0.82 in the IG and CG, respectively, showing no significant difference (*p* = 0.271) and an AUC difference of − 0.092 (95% CI − 0.255–0.071).

The mean confidence index for diagnosis based on a five-point Likert scale was 4.0 (80%) (95% CI 3.7–4.2) and 3.8 (76%) (95% CI 3.4–4.1) in the IG and CG, respectively, with no significant difference (*p* = 0.64). The mean confidence index for students’ machine use was 4.3 (86%) (95% CI 4.1–4.4) and 4.2 (84%) (95% CI 3.9–4.5) in the IG and CG, respectively, with no significant difference (*p* = 0.93). Considering image quality, no statistically significant difference was found between groups (Table 2). A significant difference was nonetheless observed in terms of machine use, with two of the examiners (surgeon and radiologist) favoring the IG (Table 2). The mean acquisition time was similar in both groups with no significant difference: 262 (+/−15) s in the IG and 255 (+/−13) s in the CG (*p* = 0.88).

**Table 2:** Quality of ultrasound image and machine use

	Examiner 1 (Surgeon)	Examiner 2 (Radiologist)	Examiner 3 (Emergency physician)
Quality of ultrasound image			
Interpretable % (*n*)			
Control group	78% (39)	72% (36)	76% (38)
Intervention group	82% (41)	78% (39)	80% (40)
*p* value	0.6171	0.4884	0.6292
Quality of machine use			
Satisfactory % (*n*)			
Control group	92% (46)	54% (27)	82% (41)
Intervention group	100% (50)	74% (37)	92% (46)
*p* value	0.0412*	0.0372*	0.1371

*Statistically significant

Regarding the three possible diagnoses (normal gallbladder, gallstones, and polyps), the IG provided 0.86 (95% CI 0.74–0.93) correct diagnoses, whereas CG gave 0.82 (95% CI 0.69–0.90). There was no significant difference between the groups (*p* = 0.59). The number of correct diagnoses per group and per patient is reported in Table 3.

**Table 3:** Number of correct diagnoses per group and per patient

Patient (diagnosis)	Intervention group *n* (95% CI)	Control group *n* (95% CI)
Patient 1 (normal)	9/10 (0.60–0.98)	8/10 (0.49–0.94)
Patient 2 (gallstone)	10/10 (0.72–1.00)	10/10 (0.72–1.00)
Patient 3 (normal)	10/10 (0.72–1.00)	8/10 (0.49–0.94)
Patient 4 (wall-echo-shadow sign)	7/10 (0.40–0.89)	6/10 (0.31–0.83)
Patient 5 (polyps)	7/10 (0.40–0.89)	9/10 (0.49–0.94)

CI: confidence interval

## Discussion

This study reported similar estimates in both groups (sensitivity 0.85 IG and 0.80 CG; specificity 0.97 IG and 0.83 CG) and a lack of significant difference for AUC (> 0.9 IG and > 0.80 CG). To our knowledge, this is the first study to evaluate practical PoCUS training using exclusively healthy volunteers according to a randomized control design. Despite its small population sample and non-clinical setting, this study reported estimates for sensitivity and specificity similar to those found in a 2011 meta-analysis, which observed pooled sensitivity and specificity of 0.90 (95% CI 0.86–0.93) and 0.88 (95% CI 0.84–0.93), respectively, for gallstone diagnosis in patients suffering from right upper quadrant pain [23]. In this pilot study, the lack of significant between-group differences suggests that the training with healthy volunteers allows students to acquire the necessary practical skills for gallbladder PoCUS assessments, similar to the skills gained from standard teaching using both pathological and normal ultrasound patterns. The study therefore supports our primary hypothesis that IG students who learn to use PoCUS on healthy volunteers can develop a comparable diagnostic accuracy to CG students. The mean confidence index above 75% for students’ confidence about their diagnosis and machine use is also promising, as it highlights that students can gain confidence in PoCUS use even without previous experience with pathological cases. Given the pilot nature of this study, these observations should nevertheless be confirmed in a larger sample of students.

Concerning the secondary outcomes, the lack of significant difference in terms of acquisition time and ultrasound image quality indicates the comparable skills of students. However, the significant between-group difference for machine use as evaluated by two of the examiners is surprising. This may be explained by the small sample size, as one student could have influenced the results. The different assessments of machine use made by the radiologist and the other two examiners may relate to how gallbladder PoCUS was taught to the students. Indeed, the technique used for gallbladder assessment with PoCUS slightly differs from the comprehensive ultrasound performed in radiology, which may explain the lower number of PoCUS considered satisfactory by the radiologist compared to the other examiners. The evaluation of image quality and machine use is nonetheless influenced by the examiners’ subjectivity, as these were not assessed using objective evaluation instruments.

The supplementary analysis highlights 20 correct diagnoses (100%) in both groups for gallstone in patient 2. The absence of patients presenting micro-gallstones or sludge is nevertheless a limitation of this study. Micro-gallstones or sludge are known to be clinically relevant, especially for migrations that result in biliary colic among others [24]. The presence of a wall-echo-shadow (WES) sign in patient 4 counterbalanced the perhaps straightforward macro-gallstone diagnosis, as WES signs are viewed as challenging in gallbladder ultrasound evaluations [25]. In the IG, 19 (95%) diagnoses of normal gallbladder were correct compared to 16 (80%) in the CG. If larger studies can show a statistically significant difference regarding the ability of students taught exclusively on normal volunteers to detect normal gallbladder, this could further support their ability to exclude the presence of gallstones, and therefore cholecystitis, in a clinical setting. Indeed, cholecystitis is known to be secondary to gallstones in 95% to 99% of cases in an emergency setting [26]. It has also been demonstrated that PoCUS can be useful to rule out cholecystitis [27]. Ruling-in and ruling-out are two distinct processes that strengthen physical evaluations and improve diagnostic approaches. Although PoCUS has long been used for ruling-in diagnosis, it should also be advocated for ruling-out. Training on healthy volunteers could help students become used to normal patterns and thus strengthen their ruling-out accuracy.

Several studies explore the feasibility of PoCUS curricula in medical schools [28, 29] and support its different applications to understand anatomy, improve physical examination, increase pathological understanding, and expand diagnosis capacity among others [30]. If larger student samples with other PoCUS teaching subjects can confirm the absence of statistical difference between students taught exclusively on healthy volunteers and those trained with pathological cases, this would facilitate the delivery of larger PoCUS practical courses, notably in undergraduate medical education. Students could even practice on each other, which would avoid the need for volunteers with pathologies. This could also strengthen bedside physical examination before students begin clinical rotations by enhancing PoCUS skills, improving students’ toolkit for clinical evaluation, and thus helping bedside supervision.

### Strengths and limitations

The randomized control design of this pilot study and its setting is a strength, as the intervention was the only variable. Indeed, the careful selection of volunteers for the practical sessions ensured the absence of between-group differences in terms of age, weight, height, and BMI. Thus, PoCUS difficulty was similar for all students. One possible limitation was that the instructors were not blinded, which could have influenced the training of each group. This bias was nevertheless limited by the strict teaching protocol in which the same PoCUS information was given to all students.

The study duration over 2 months could have led to a breach of the non-disclosure agreement for the students or instructors. However, it is unlikely that students practiced gallbladder PoCUS outside of the study setting, as there is no ultrasound laboratory in our university, and students did not have clinical rotations during the study period. Indeed, students were unaware of the study outcomes and evaluation process in advance. The instructors were mostly from external hospitals and were not in contact with the students.

The study coordinator decided to include fifth-year medical students in the study, which could be seen as a limitation. Indeed, several publications focus on PoCUS curricula for undergraduate medical education, with some American authors advocating the teaching of gallbladder PoCUS in the second year of medical school, equivalent to the fourth year in Europe [31].

One strength of this study was the interval between the practical sessions based on a recent review for medical education [21]. Unfortunately, the study setting did not allow for a longer period of time before the evaluation day, although students demonstrated satisfactory practical knowledge retention after 2 months.

Another strength was students’ random allocation to different schedules over the two evaluation days, as it prevented the examiners from extrapolating their group allocation. For the evaluation, students were given a random number and asked not to interact with the examiners. Examiners were blinded to the students’ diagnoses so as not to be influenced by their diagnostic performances when evaluating image quality and machine use. Students were nevertheless asked to fill in a separate document with their diagnosis and confidence index of each PoCUS, which prevented them from changing their diagnosis after completing the examination; this is another strength of the study setting.

On the evaluation days, the order of the volunteers did not change. All volunteers fasted for at least 3 h before the first gallbladder evaluation, although they were allowed to drink water throughout the evaluation due to its long duration. This could be a limitation, as could the time of day, which would have influenced the amount of gas in the patients’ guts and thus impacted the PoCUS assessment. This bias was nevertheless reduced by students’ randomization for the evaluation, which possibly homogenized the varying difficulty of PoCUS between groups.

The absence of a clinical setting was a limitation of this study, although the lack of clinical stress could have favored students’ concentration and diagnostic accuracy. This parameter was, however, counterbalanced by the stress of the evaluation day due to the presence of three renowned examiners during the PoCUS assessment.

## Conclusion

This pilot randomized control trial shows that medical students trained on exclusively healthy gallbladders can become proficient in assessing gallbladders for gallstones using PoCUS. These findings could form the basis of prospective randomized trials to evaluate students’ performances for the diagnosis of pathological PoCUS signs, even though they developed their skills on volunteers unlikely to have the targeted condition. This could facilitate the organization of large practical PoCUS sessions as part of the undergraduate curricula without the requirement for initial training in clinical settings. If confirmed by a larger sample of students, the encouraging sensitivity results found in this study could promote the development of the rule-out capacity of bedside PoCUS.

## Supplementary Information

**Additional file 1.** PoCUS teaching chart.

## Data Availability

The datasets used and analyzed during the current study are available from the corresponding author on reasonable request. All data generated or analyzed during the study are included in this published article.

## Abbreviations

AUC: Area under the curve
BMI: Body mass index
CG: Control group
CI: Confidence interval
IG: Intervention group
PoCUS: Point-of-care ultrasound
ROC: Receiver operating characteristic
WES: Wall-echo-shadow
