# Supplementary material for: Is gallbladder PoCUS diagnostic accuracy accessible to medical students after PoCUS training exclusively on healthy volunteers? A pilot randomized control trial
Source: Ultrasound J. 2023 Apr 10;15:18. doi: 10.1186/s13089-023-00317-6 (PMC10086079; doi:10.1186/s13089-023-00317-6)
Supplement: Supplementary file 1 — Additional file 1. PoCUS teaching chart. [file 13089_2023_317_MOESM1_ESM.pdf]

Gallbladder PoCUS messages

Ultrasound probe use

- ☐ Curvilinear probe
- ☐ Abdominal pre-set
- ☐ Adequate depth and gain

Finding the gallbladder

- ☐ Adequate probe holding
- ☐ Intercostal and sub-costal view of the gallbladder
- ☐ Longitudinal and transverse view of the gallbladder
- ☐ Fanning through the entire gallbladder
- ☐ Improving views by left lateral position and holding a deep breath

Normal gallbladder

- ☐ Ultrasound aspects
  - ☐ Fundus – body - neck
  - ☐ Anechoic content
  - ☐ Hyperechoic and thin wall
  - ☐ Posterior hyperechoic zone
  - ☐ No posterior acoustic shadowing
  - ☐ Edge shadowing artifact
- ☐ Other normal aspects
  - ☐ Collapsed gallbladder
  - ☐ Gallbladder septation
- ☐ Pitfalls
  - ☐ Stomach and duodenum
  - ☐ Digestive structures or gas
  - ☐ Edge shadowing artifact

Pathological Gallbladder

- ☐ Gallstones
  - ☐ Unique or multiple
  - ☐ Micro or macrolithiasis
  - ☐ Hyperechoic–Glittering
  - ☐ Posterior acoustic shadowing
  - ☐ Move with position changes
  - ☐ No movement if impacted in the neck
  - ☐ Wall Echo Shadow Sign
- ☐ Sludge
- ☐ Polyps
